# An exploratory assessment of the legislative framework for combating counterfeit medicines in South Africa

**DOI:** 10.1186/s40545-021-00387-8

**Published:** 2022-01-05

**Authors:** R. J. Moshoeshoe, G. M. Enslin, D. R. Katerere

**Affiliations:** Department of Pharmaceutical Sciences, Tshwane University of Technology, Private bag X640, Pretoria, 0001 South Africa

**Keywords:** Counterfeits, Substandard medicines, South African regulations, COVID-19 pandemic, Pharmaceutical policy, Regulation of medicines, Public health

## Abstract

**Background:**

Substandard and Falsified (SF) medical products are a growing global concern. They harm the individual patient, the healthcare system and the economy. The World Health Organisation (WHO) has highlighted contributing factors globally: insufficient national medicine regulation, poor enforcement of existing legislation, weak stakeholder collaboration and the rise of novel viruses, such as the COVID-19. The study aimed to assess the legislative and policy framework and institutional relationships governing pharmaceuticals and anti-counterfeiting strategies.

**Methods:**

The study was explorative and consisted of two phases. The first phase was between 2016 and 2017. It looked at document analysis (annual reports and press releases from 2011 to 2016) from government institutions involved in medicines regulation and law enforcement for SF seizure reports between 2004 and 2017. The second phase was between 2016 and 2018 through in-depth semi-structured interviews (seven in total) with selected stakeholders.

**Results:**

First Phase—the data collected and reported by various departments was sporadic and did not always correlate for the same periods indicating, a lack of a central reporting system and stakeholder collaboration. In South Africa, counterfeiting of medicines mainly involves the smuggling of non-registered goods. The most common counterfeit items were painkillers, herbal teas, herbal ointments, while some were medical devices. Furthermore, Customs identified South Africa as a transhipment point for SF infiltration to neighbouring countries with less robust regulatory systems. Second phase—interview transcripts were analysed by thematic coding. These were identified as the adequacy of legislation, institutional capacity, enforcement and post-market surveillance, stakeholder collaboration and information sharing, and public education and awareness.

**Conclusion:**

Document analysis and interviews indicate that South Africa already has a national drug policy and legislative framework consistent with international law. However, there is no specific pharmaceutical legislation addressing the counterfeiting of medicines. Law enforcement has also been complicated by poor stakeholder engagement and information sharing.

**Supplementary Information:**

The online version contains supplementary material available at 10.1186/s40545-021-00387-8.

## Introduction

Counterfeit medicines, are becoming a global public health problem in both developing and developed countries [1]. Counterfeit medicines have been found in street markets, legal supply chains like, health care facilities as well as illegal or unregulated websites [2, 3]. The COVID-19 pandemic has led to an increase in the supply demand for pharmaceutical products, including vaccines [4]. This COVID-19-induced demand for health products has also been exploited by organized crime groups specifically in developing countries in Africa [5, 6]. The United Nations Office on Drugs and Crime (UNODC) reported an increase in trafficking incidents during the pandemic at African seaports. For instance, Lome (Togo), Cotonou (Benin) and Mombasa (Kenya).

According to United Nations [7] these crime groups manufacture, traffic and sell a variety of products from diagnostic testing kits to treatments to preventative measures, such as sanitisers, vaccines and Personal Protective Equipment (PPE). From the beginning of January through mid-April of this year, COVID-19-related scams in the USA totalled approximately US$ 13.4 million in fraud. Furthermore, a total of 1541 cyberattacks related to COVID-19 were detected in the United Arab Emirates, including 775 malware threats, 621 email spam attacks, and 145 URL attacks. In Thailand, about three hundred thermometers were seized in Thailand after being trafficked through three other countries. Similarly, thermometers that did not meet EU regulations were found in Italy [7].

While in the United Kingdom, the Medicines and Healthcare Products Regulatory Agency (MHRA) released figures revealing that approximately 3.5 million unlicensed erectile dysfunctional pills, worth more than £10 million, were seized in the UK in 2019 [8].

The counterfeiting business is highly lucrative, and because it is illegal [9], counterfeiters do not register their dealings and easily evade tax [10]. Companies that deal with pharmaceuticals have been hit the hardest and risk loss of their reputation. Consequently, they may also have to take legal action to protect their brands [11]. In addition, the loss of profits and high legal fees have led to the reduction of research and development investments and job losses in this sector [12]. A study conducted by the European Union Intellectual Property Office in the European Union analysed the cost impact of counterfeit medicines on the pharmaceutical industry to be €10.2 billion euros yearly and an additional value of about €7.1 billion euros from related sectors. It was speculated that these losses in profits could have been responsible for at least 37 700 job losses annually [13].

Scammers and fraudsters have increased significantly during this pandemic. Prior to the COVID-19 global crisis, the Internet offered consumers cheap, otherwise “stigmatised” and controlled medicines without prescription [14]. In addition to so called “miracle cures” the Internet now offers cheap sanitisers, thermometers and surgical masks deceiving consumers even more [15].

A major problem with illegal internet pharmacies and traders is that they usually conceal their real identity, while they ship medications and products with questionable quality and traceability across borders [16]. Since counterfeiters use illegal clandestine channels to introduce these into the market, it is difficult to quantify the extent of proliferation [17]. According to Mackey and Liang [18] the Internet has made counterfeit medicines a transnational and an international crime.

The South African Health Products Regulatory Authority (SAHPRA) is considered mature and stringent [19] when compared with other counterparts in the Southern African Development Community (SADC) region, such as Lesotho, which has no existing regulatory authority [20]. Many developing countries do not have a mature regulatory framework that can take preventative measures due to lack of capacity, technical expertise and financial resources to undertake surveillance [21].

A recent study assessing medicine quality in the South African supply chain showed that although no counterfeited products were identified, only about 55.4% (173/312) of the samples met the US pharmacopoeia standards for quality [22]. Many of them failed the visual inspection tests, and 5.4% failed the dissolution tests. Demonstrating that perhaps regulatory activities tend to focus more on pre-market authorisation than on Post Market Surveillance (PMS) and pharmacovigilance. Another study by Patel et al. [23] examining the perceptions of stakeholders on drug quality in South Africa also highlighted similar views.

On this note, this study aims to contribute to what is known about the regulation of counterfeit medicines in the South African context, the challenges and the opportunities that exist to better combat strategies.

## Materials and methods

- The second phase of the study was conducted from 2016 to 2018. Prior to conducting the study, Ethics Approval was granted by the Faculty Committee for Postgraduate Studies and the Research Ethics Committee of Tshwane University of Technology on the 28 July 2016. The Ethics Committee for Research Ethics reference number given was FCRE: 2016/05/001 (3) (SCI).
- Invitations were sent to all seven stakeholders who participated in the study and informed consent were given and signed by all before commencement of data collection.
- The quality of data collected was ensured by following the “Principles of Trustworthiness”. These were established by ensuring credibility, confirmability, transferability and dependability as prescribed by Anney [24] See Additional file 7 for how the researcher ensured the quality of data.

### First phase of study

The first phase of the study—document analysis was conducted on public records of governmental institutions. These were press releases and annual reports for the periods between 2004 and 2017. The focus of the analysis was on reported incidents of counterfeit products (SFs) and seizure of such goods by border authorities. The researcher  also considered reports of SFs by regulatory authorities. It is important to note that the reporting on SF seizures varied from department to department even for the same period.

### Inclusion criteria

The search engines used were *Google* and *Google Scholar* and the websites visited were government databases and international advocacy group databases (see Additional file 6) for the list of databases). The keywords used for the search were: Counterfeits, substandard medicines, South African regulations, pharmaceutical policy, regulation of medicines, public health or synonyms. Reports only in English were considered.

### Exclusion criteria

Data on other types of counterfeited products other than pharmaceuticals, records on counterfeit pharmaceutical products from other countries other than in South Africa and annual report records before the financial period of 2011. Reports not in English were excluded.

### Data collection of press releases and annual reports

The data from press releases and annual reports were collected using a data collection tool (Additional file 5) categorised into the following: *type of seizure*, *type of offence*, *place/period of seizure*, *the quantity of seizure* and *the net worth/value of products*.

### The second phase of study

The second phase was conducted by interviewing seven key stakeholders (see Table 1). These were identified as, the customs and border control, the police service, the national trade regulatory authority, Intellectual Property Rights (IPR) attorneys, the professional pharmacy council, the national medicines regulatory authority, and the national pharmaceutical procurement depot. The interviews took place between 2016 and 2018.

**Table 1:** Stakeholders for the study (*n* = 7)

Organisation	Experience
Intellectual Property Experts	Brand Protection
Law Enforcement (NMRA): SAHPRA/MCC	Inspections of counterfeit and illegal medicines
Law Enforcement: SAPS	Investigations of counterfeit goods Policing against criminal activities
Law Enforcement: SARS (Customs)	Inspections and border control
Statutory Professional Council: SAPC	Inspection and registration of Pharmacies and pharmacists
Wholesaling: Provincial Pharmaceutical Depot	Procurement of Medicines
Industry Compliance: DTI/CIPC	IPR enforcement Inspections

### Sample selection and recruitment

Purposive and snowball sampling was used to select study respondents. Respondents were chosen based on their experience and expertise in the subject matter. Persons and organisations involved in law enforcement agencies (police, customs, the NMRA, industry regulators, wholesaling and legal fraternity) dealing with procurement of pharmaceutical products or the combat of in their line of work in South Africa were selected.

### Data collection

Prior to data collection, the study participants signed informed consent forms (see Additional file 5). Seven interviews were done on selected respondents. An interview guide was adapted from a World Health Organisation data collection tool [25] which was previously designed to provide a review for drug regulatory systems (see Additional file 2). Follow-up telephonic and email interviews were done with respondents, where clarity was needed. Principles of anonymity and trustworthiness were applied during the interview process (Additional file 8).

Only handwritten interview transcripts were used, because interviewees requested not to be recorded on audio devices. Therefore, there were no audio records used for this study.

### Data analysis

Data analysis was done in three stages, by line by line coding of the findings from the interviews and the organisation of those ‘free codes’ to construct ‘themes’ [26]. The following steps were followed during the coding process:

i) The evaluation of concrete evidence which involved reading through interview transcripts and notes and reflection;
ii) Thematic analysis based on similarities and differences between respondent responses for the same questions, then data was organised into themes and then sub-themes by the aid of Microsoft Word (Macros add-in *DocTools*);
iii) Interpretation of these themes and subthemes was the final process.

## Results of document analysis

### Gaps in the legislation

The incident reports (see Table 2) revealed that the most common type of offence or contravention of the law was due to unregistered or expired medicines, false claims of “herbal supplements”, sale of controlled substances without prescription and licenses (Medicines and Related Substances Act 101/65, Section 18 and 22C). These offences are all in contravention of the laws governing the regulation of medicines in South Africa [27].

**Table 2:** Data mostly of local brands that haven counterfeited and unregistered products

Type of pharmaceutical product or case report	Type of offence	Manufacturer affected	Place/period of seizure	Seizure units/net worth	Data source
Adlam case	Expired medicines	Unspecified	2004	R 30 million	SAHPRA, 2017
Vally Case	Expired and unregistered	Unspecified	2007	R 130 million	SAHPRA, 2017
Ephedrine transhipment to Swaziland	Unregistered medicine. No import permit	Unspecified	2008	Unspecified	SAHPRA, 2017
Zambuk Ointment	Counterfeit	Bayer Healthcare	2011	350 000 tins	SAHPRA, 2017
Grandpa Batch no:	Counterfeit	Glaxo-Kline & Smith		Unspecified (referred to as millions of Rands (ZAR)	SAHPRA, 2017
309339			2011		
314020			2017		
Simply Slim Case	False Advertisement and labelling Claim: 100% herbal Contents: 27 mg Subutramine	Unspecified	2010	Unspecified	SAHPRA, 2017
Viagra	Counterfeit	Pfizer	2010–2014	Unspecified	SAHPRA, 2017
Cialis	Unregistered	Eli Lily & Co	2010–2014	Unspecified	SAHPRA, 2017
Flu Vaccine	Unspecified	Pfizer	2015	Unspecified	SAHPRA, 2017
Vicks Vapo Rub 12 g tin	Counterfeit	Procter & Gamble trading	2017	38 Count Packages	SAHPRA, 2017
Skin lightening Creams	Transhipment by well-known importer for diversion of goods	Unspecified	Johannesburg	9 141 boxes	DTI, 2017
Medical drips (Fresenius and Kabi)	No export permit Destined for Mozambique	Unspecified	Kempton Park (Pomona)	4 pallets	DTI, 2017
Herbal teas and creams	Falsely labelled	Unspecified	Pretoria (Sunnyside)	Unspecified	DTI, 2017
Slimming Tea	Unregistered importer	Unspecified	Durban	942 cartons or 885 packages	DTI, 2017
Ampilox		Unspecified	Durban	1 900 vials	DTI, 2017
Illegally manufactured (API)	Illegal manufacturer GMP Non-compliance	Unspecified	Cape Town Int. Mailing Centre	Unspecified	DTI, 2017

Source: DTI (2017); SAHPRA (2017)

Other offences included illegal manufacturing of Active Pharmaceutical Products (API), contravention of Good Manufacturing Practice requirements, illegal importing of goods (Medicines and Related Substances Act 101 of 1965, Section 15C; Customs and Excise Act 91 of 1964; Pharmacy Act 53 of 1974, Section 22), and false advertising and falsified labelling of products (Medicines and Related Substances Act 101 of 1965, regulation 12).

### Enforcement and post market surveillance

Based on the review of the annual reports, it was apparent that data collection and reporting on SFs were weak across departments, indicating that counterfeiting of medicines was not urgent as counterfeiting of tobacco, clothing, and even electronics (South African Revenue Service annual report 2014/15). There was inadequate information on counterfeit medicine seizures for the stipulated period (2004–2017) and the data found varied considerably between government agencies. This is indicative that data collection on counterfeit medicines is not systematic, information sharing is poor and law enforcement activities are not coordinated.

Although efforts were made to enforce IPRs and strengthen border security, SFs were not always given top priority as a serious public health problem. Furthermore, it is not clear what role the NMRA played in coordinating the fight against SFs in South Africa. The trade and industry department (DTI) and customs division (SARS) were the two leading agencies in the combat against SFs. It is important to also note that, Adverse Drug Reactions (ADRs) were only briefly mentioned in the annual reports of the Department of Health. According to respondents, the lack of capacity, however, has prevented the NMRA from acting on ADRs, which are the responsibility of the pharmacovigilance arm of the NMRA.

Furthermore, the Department of Trade and Industry's (DTI) arm, the Companies Intellectual Property Commission (CIPC) in a joint enforcement effort with the private sector, planned to issue Internet Service providers (ISPs) with notices for allowing the sale of unregistered, and potentially counterfeit medicines online.

### Institutional or organisation capacity

As shown in the Department of Health's (DoH) annual reports, the former MCC did not have its own budget. The work was funded through proceeds from product registrations fees which was not sufficient to cover the complex nature of enforcement work. Although, the NMRA was the inspectorate arm of the Department of Health, enforcement activities were not included in the annual reports, but only information on product registrations was mentioned.

In general, departments reported mostly on goods seized for not complying with import processes rather than for the contravention of pharmaceutical requirements and regulations and/or causing harm as a result. The review also revealed that the perception that issues affecting medicine quality and supply chain integrity in South Africa were not attributed to counterfeited medicines but rather unregistered products to be partly true. This similar observation was also reported in the study by Patel et al. [23] which looked at the perception of key players involved in the medicines value chain by respondents. Most seized goods were unregistered products which are almost always substandard. A single incident of raw materials (API) being illegally manufactured in Durbanville, Cape Town, was reported. It was not clear whether the product was falsified or substandard but it was found at the mailing center destined for export to a country that was not mentioned in the seizure or incident report (see Table 2).

## Results of interviews

A number of challenges in the combat of SFs were identified in the study through views and perceptions of respondents. The main themes that emerged were: adequacy of legislation, institutional/organisational capacity, enforcement and market control, stakeholder collaboration and information-sharing and education and awareness (see Table 3) shows an overview of the analytical framework used in the thematic analysis.

**Table 3:** Summary of the interview results and interpretation thereof based on the thematic analysis

Interview main themes	Sub themes	Implications
Adequacy of Legislative	Strong legislation, however, gaps exist	Absence of a pharmaceutical anti- counterfeiting strategy
	Pharmaceutical regulation focuses on market authorisation and GMP, not counterfeit medicines	Inadequate policing powers for NMRAs and customs
	Pharmacovigilance focuses on Adverse Drug Reactions not reporting of counterfeit incidents	Lax penal sanctions
	Absence of a legal mandate to combat counterfeit medicines	Lack of harmonisation of existing laws and functions of the NMRA
	Poor implementation of the law	Poor prosecution outcomes
		Due to challenges with the judiciary and tedious requirements lodging a criminal case
Institutional/organisational capacity	Inadequate Resources	Limited human resources and field training
		Budget constraints
		Shortage of accredited testing and QA facilities
	Legal Challenges	Lack of political will
	Market controls and Inspections	Weak penalties
		Lack of harmonisation of existing legislation
		Corruption
Enforcement and Post Market Surveillance		Weak enforcement
	Protocols and Procedures	No Single Point of Contact (SPOC) for reporting counterfeit incidents
	Managing conflict of interest	Poor Post Market Surveillance
		Lack of online regulatory controls (see Tables 5 and 6)
Stakeholder Collaboration and Information Sharing	Poor National Collaboration	Ineffective interagency platforms and communication
		The duplicity of function due to competing mandates in enforcement agencies
	Regional and International Collaboration	Poor regional interaction on Counterfeit medicine issue
		Effective joint international initiatives (WCO-INTERPOL)
Public Education and Awareness	Awareness campaigns	Lack of transparency between NMRA and pharmaceutical industry on information sharing
		Lack of public awareness campaigns. Not a priority area in health promotion goals or IPR infringements
		A lax approach to dangers of counterfeit medicines by departments in comparison to other counterfeit good

### Gaps in the legislation

**Fig. 1:**
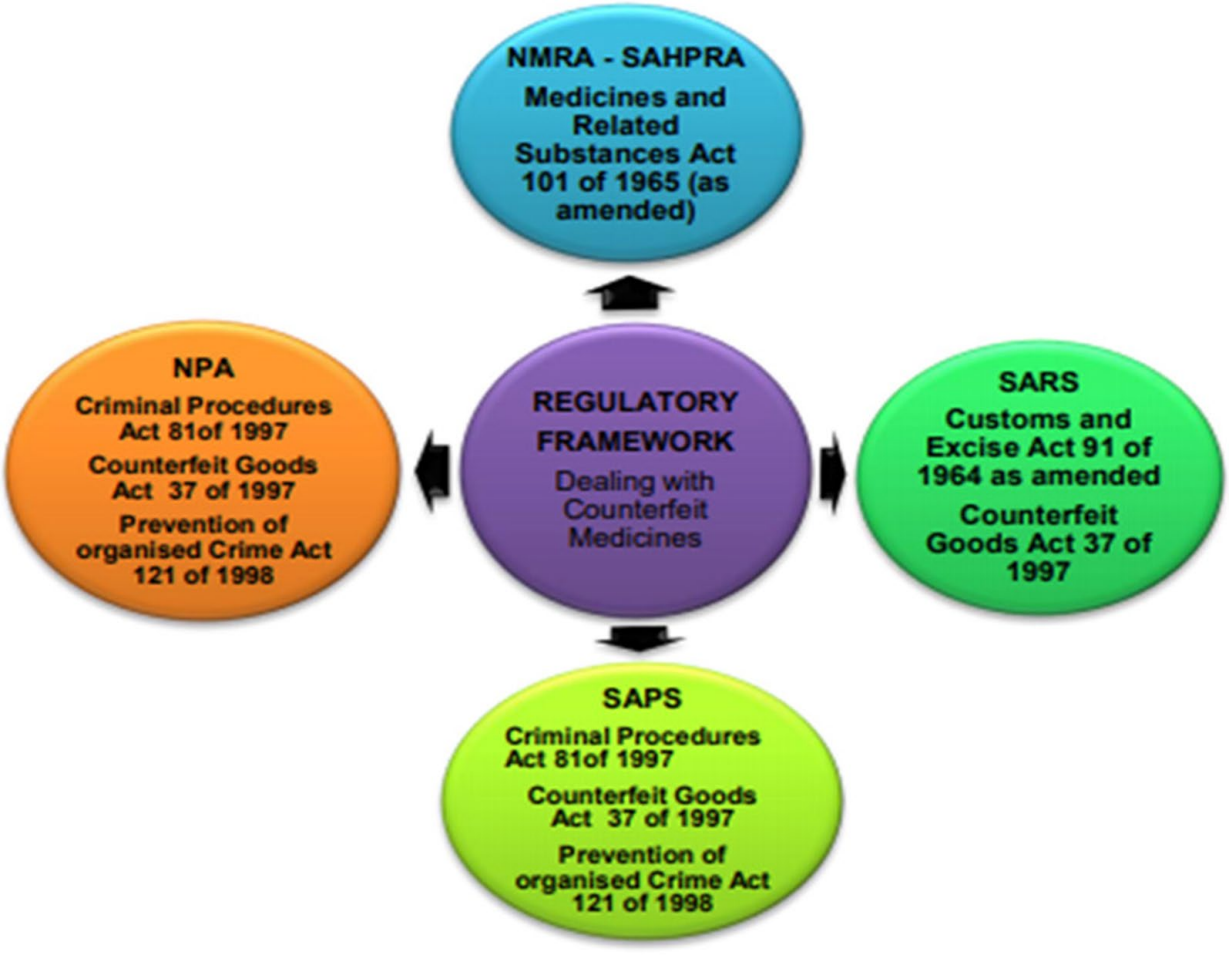
South African regulatory framework and stakeholders responsible for enforcement. This shows the legislation involved and the relevant departmental/agency

Respondents perceived that there was a stringent regulatory environment for pharmaceuticals; however, it was not clear or adequate on how to deal with the issue of SFs. The study revealed that the main pieces of legislation responsible to combat of SFs are the Counterfeit Goods Act 37 of 1997, the Medicines and Related Substances Act 101 of 1965, the Customs and Excise Act 91 of 1964, the Criminal Procedures Act 51 of 1997 and the Prevention of Organised Crime Act 121 of 1998 [28–30] which required joint stakeholder engagement to implement (see Fig. 1). See also Table 4 for the practical implications of each law. Many of the applications of these laws overlapped in the inspection function (see Fig. 3) of regulating pharmaceuticals.

**Table 4:** How the legislation cited in the manuscript can be applied in the prosecution process

Data source/date published	Type of legislation	Penalty/sentences	Stakeholders in law enforcement	Practical implications
Government Gazette	Counterfeit Goods Act 37 of 1997, Sect. 19(1) TRIPS agreement article 61 Provisions	*1*^*st*^* offence:* ≥ *R5000 per item or with imprisonment not exceeding 3* *years*	SAPS (Commercial Crime Unit) DTI -CIPC (IPR protection) SARS (Customs)	This law focuses primarily on IPR infringements of all patented or trademarked products including medicines
01-Oct-97		*2nd offence*: ≥ *R10 000 per item with 5* *year imprisonment*		
Government Gazette	Medicines and Related Substances Act 101 of 1965	Offences relating to sale of medicines (S14), false labels & advertisements (S18), out of specifications, controlled substances and licenses (22C), sale and purchase by wholesalers (22H):	SAHPRA (National Medicines Regulatory Authority)	The law focuses on licensing pharmacies, dispensing practitioners, labelling requirements, the manufacturing, sale, and distribution of medicines, the sale and distribution of narcotics, the sale of medicines requiring special permits, as well as permits for the movement of medicines in and out of South Africa. There is no specific provision regarding the sale of medicines online
19 June 1965		Liable to a fine or imprisonment not exceeding 10 years		
Government Gazette 23 July 2014	Customs and Excise Act Section 113A, Section 5, Section 4(1)	Customs duties & taxes	SARS (Customs)	This law outlines requirements for the movement of medicines across national borders, wholesale licenses, infringement of intellectual property rights, and illegal substances through the Harmonised System of Tariffs that identify high-risk goods
Government Gazette 04 December 1998	Prevention of Organized Crime Act 121 of 1998	*Proceeds of crime:* ≥ R100 million or imprisonment not exceeding 30 years	NPA (judiciary) SAPS (Commercial Crime Unit)	This law focuses on the penalties for money laundering and proceeds made from organised crime. If trafficking of counterfeited or falsified medicines involves organise crime, then this law will be applied
		Section 8(1): Criminal gang activities:		
		Imprisonment: Minimum: ≥ 3 years. Maximum: ≥ 8 years		
		*Asset forfeiture* (Section 30)		

 There was also a perception that South Africa was not a source of origin for counterfeit medicines and that it was not a big problem on the market.

“*Section 113A, Section 15 and Section 4(1) of the Customs Act, the Counterfeit Goods Act 37 of 1997 and the Medicines and Related Substances Act cover the powers of customs officers and inspectors to search, seize and detain to regulate importation and exportation of medicines.” Respondent 3*.

“*Medicines can only be allowed into South Africa through Cape Town (sea and air), Durban (sea and air), Port Elizabeth (air and sea) and OR Tambo international airport either way they are sent back to the country of origin.” Respondent 2*.

“*Yes, there are laws we can use though not specifically for pharmaceutical crime, but they focus more on counterfeited trademarks and infringement of copyrights of known clothing brands, electronics and DVDs.” Respondent 7*.

Policing of SFs was often associated with the unauthorised sale of medicines rather than falsified medicines by respondents from the NMRA. As cited by a respondent from law enforcement: *“We do not have a problem with counterfeit medicines only unauthorised medicines.”*

Some respondents cited gaps in the legislation and called for a more specific legislation to address the complexities encountered in handling pharmaceutical crime.

The many complexities included the definition of *counterfeit medicine* which catered for IPR infringement but not necessarily the safety and quality of medicines.

The main gaps in the current legislation were identified as:

i) The lack of harmonisation of existing laws which created loopholes in prosecutions;
ii) Jurisdictional limitations between stakeholders made it difficult to address the technical aspects of prosecutions that required expertise from the judiciary in handling pharmaceutical crime;
iii) Penal sanctions stipulated were a “manageable business” cost and not deterrent enough (fines and light prison sentences);
iv) Weak regulation of the sale of online medicines. The law focused on false advertising and labelling. There was no allocation in the law (the Medicines and Related Substances Act 101/1965) for requirements on online pharmacy registrations or operations (see Table 5 for a review of the legislation regarding operating online pharmacies);
v) The absence of a specific anti-counterfeit legislation or policy to mandate stakeholder engagement and enforcement activities;
vi) Poor prosecution outcomes due to lack of cross skill expertise in handling pharmaceutical crime cases and lack of political will to prosecute cases of this nature.

**Table 5:** Review of contravention of the law by three online pharmacies (see Table 6) identified during the study

Legislation review	Website review
Scheduled medicines/authorised persons: The websites were open to the public so any person can order scheduled medicine being offered (Scheduled 4 unregistered Biological injectable products are being offered)	Section 22A of Act 101 clearly states—“(…) no person shall sell, have in his or her possession or manufacture any medicine or Scheduled substance, except in accordance with the prescribed conditions.” Furthermore, Section 14 (5) stipulates who may possess or sell Scheduled medicines and under what conditions
Registration of medicines: Some of the Schedule 4 medicines (biological injectable) offered were not yet registered in South Africa, i.e. Xeomin; Bocouture; Azzalure; NeouroBloc etc.	Section 14 of Act 101 clearly states—“(…) no person shall sell any medicine which is subject to registration by virtue of a resolution published in terms of subsection (2) unless it is registered.”
Labelling requirements: Products advertised do not comply with local labelling requirements	Regulation 8 of Act 101 clearly stipulates how scheduled medicines sold in South Africa should be labelled. The website offers non-English labelled BOTOX (again Scheduled 4 Biological medicines are being offered direct to the Public)
Pharmacovigilance: No local South African contact person or site was provided on the websites. There was no indication on how adverse drug effect would be handled—an issue of public health safety	To satisfy the requirements of Regulation 37 on Adverse Drug Reactions
Pricing requirements: Products were offered and sold at higher prices than the approved Department of Health Single Exit Prices (SEP)	Section 22G of Act 101 clearly states the following under heading Pricing Committee 3(b)—“(…) no pharmacist or person licensed in terms of Section 22C (1)(a) or wholesaler or distributor shall sell a medicine at a price higher than the price contemplated in paragraph (a).”
Procurement requirements (warehousing/storage): some of the products were cold chain products—so it is unclear under what conditions these products are being shipped—a major quality concern	It is difficult to verify if GMP and GWP standards were maintained
Licensing of pharmacies: Some of the companies were not registered importers: e.g. Monarch Medical Supplies is not an authorised importer. All three websites were not registered with the SAPC	Section 22C of Act 101 clearly states the following on Licensing: “(…) no manufacturer, wholesaler or distributer referred to in subsection (1) (b) shall manufacture, import, export, act as a wholesaler of or distribute, as the case may be any medicine unless he or she is the holder of a license contemplated in the said.” subsection.” Regulation 12 of the Act states the conditions required to be able to import medicines (permits)

### Institutional resources

All respondents indicated that limited resources contributed to the weak enforcement efforts to combat SFs. The main resources mentioned were human resources, financial backing and testing facilities.

#### Limited human resources

Respondents cited that there was a staff complement of eight inspectors assigned to perform inspection on Good Manufacturing Practice (GMP) and Good Wholesale Practice (GWP). While only two were reported to be assigned to cover inspections in only two ports of entry due to high traffic, OR Tambo international airport and the Durban Harbour.

*“Only eight inspectors are doing GMP inspections and only two pharmacists in the law enforcement unit at the NMRA. Two additional inspectors cover Durban, OR Tambo airport and Cape Town mainly and the other ports of entry occasionally.” Respondent 3*

#### Financial backing

Financial backing is an important component in the work of enforcement agencies. Limited access to funding for pharmacovigilance and post market surveillance work was a common factor cited by all respondents. One of the respondents showed that the NMRA was not in good financial standing, and therefore, enforcement efforts focused on pre-market authorisations.

#### Pharmaceutical testing facilities

Our study found out that the NMRA did not have its own laboratory testing facilities but outsourced these in the past.

“*It would be helpful to have a testing device like the handheld Truscan or a mobile laboratory toolkit that can detect the contents of illegal medicines at points of entry like the ones used in other countries by customs agencies.” Respondent 3*.

One respondent cited that the provision of an in-house testing facility would be helpful in re-testing all medication procured at the pharmaceutical depot.

“*We would benefit greatly from in-house quality testing but currently we do not have a testing facility. We have to send samples to a government lab in the Western Cape and it takes time to get results.” Respondent 2*.

In retrospect, other medicines’ regulators, such as the Medicines Control Authority of Zimbabwe (MCAZ) [31], the National Agency for Food and Drug Administration and Control (NAFDAC) in Nigeria [32] and the Food and Drug Administration (US FDA) in the United States [33] and the Central Drugs Standard Control Organisation (CDSCO) in India [34] have their own laboratories.

Notably, the Medicines and Related Substances Act 101/1965 states that there are seven designated ports of entry for the movement of pharmaceuticals [27]. A respondent cited that only two inspectors were available for this purpose, leaving the other designated ports vulnerable to potential smuggling of SFs. This is a notable risk especially during high import volumes like during the COVID-19 pandemic.

### Stakeholder collaboration and information sharing

Medicine governance in South Africa, has at least six key institutional stakeholders in ensuring that safe medicines are available. These are, the pharmaceutical companies (importers, multi-national companies and local producers), the medicine regulator (SAHPRA/MCC), Department of Police (national, provincial and municipal), the South African Revenue Services (customs and border division), Department of Health, Department of Trade and Industry, the CIPC (intellectual property protection) and the Department of Justice (national and provincial prosecutors). Stakeholder collaboration and information sharing remain an important key to the combat of SFs for obvious reasons already outlined.

In dealing with counterfeit medicine cases, all study respondents agreed there was some coordination between certain agencies and not others but these working relationships had challenges. All respondents agreed that cooperation and open communication channels were essential for the smooth and effective  functioning of the agencies and  that enforcement efforts needed to be more coordinated. However, it was unclear which agency was to lead such a coordination effort.

## Discussion

The study shows that the South African legal framework is compatible with international standards. However, there is no comprehensive strategic framework for dealing with counterfeit and substandard medicines. As a result, the pharmaceutical value chain and the health care system are compromised.

It appears that counterfeit medicines are not given the same priority as other public health issues  such as the counterfeiting of tobacco. The Department of Health has identified clear priority areas which include, the prevention and treatment of HIV/AIDS, tuberculosis (TB), non-communicable diseases, tobacco use and promoting healthy lifestyles. Notably, each priority area has a policy and implementation strategy. As a result of their cross cutting impact, SFs,  should be receive the same attention and concern. They affect patient treatment outcomes, public health safety, household income if a patient dies or has prolonged illness. In addition to affecting patient treatment outcomes and the potential loss of household income if a patient dies or has prolonged disease, SFs also undermine the healthcare system in various ways. Two of them are by costing taxpayers more money to procure expensive medicines and tax evasion, further crippling the economy.

### Gaps in legislation

The subsequent result of not having an anti-counterfeiting strategy is that there is no guiding framework to facilitate the enforcement of the law. Objectives are not always clear and stakeholder responsibilities are not outlined.  This results in a lack of accountability among stakeholders and the NMRA. As well as clearly assigned leadership responsibilities for enforcement efforts. It is important to get total buy-in by all stakeholders involved in the regulation of medicines to ensure working together to combat SFs.

**Fig. 2:**
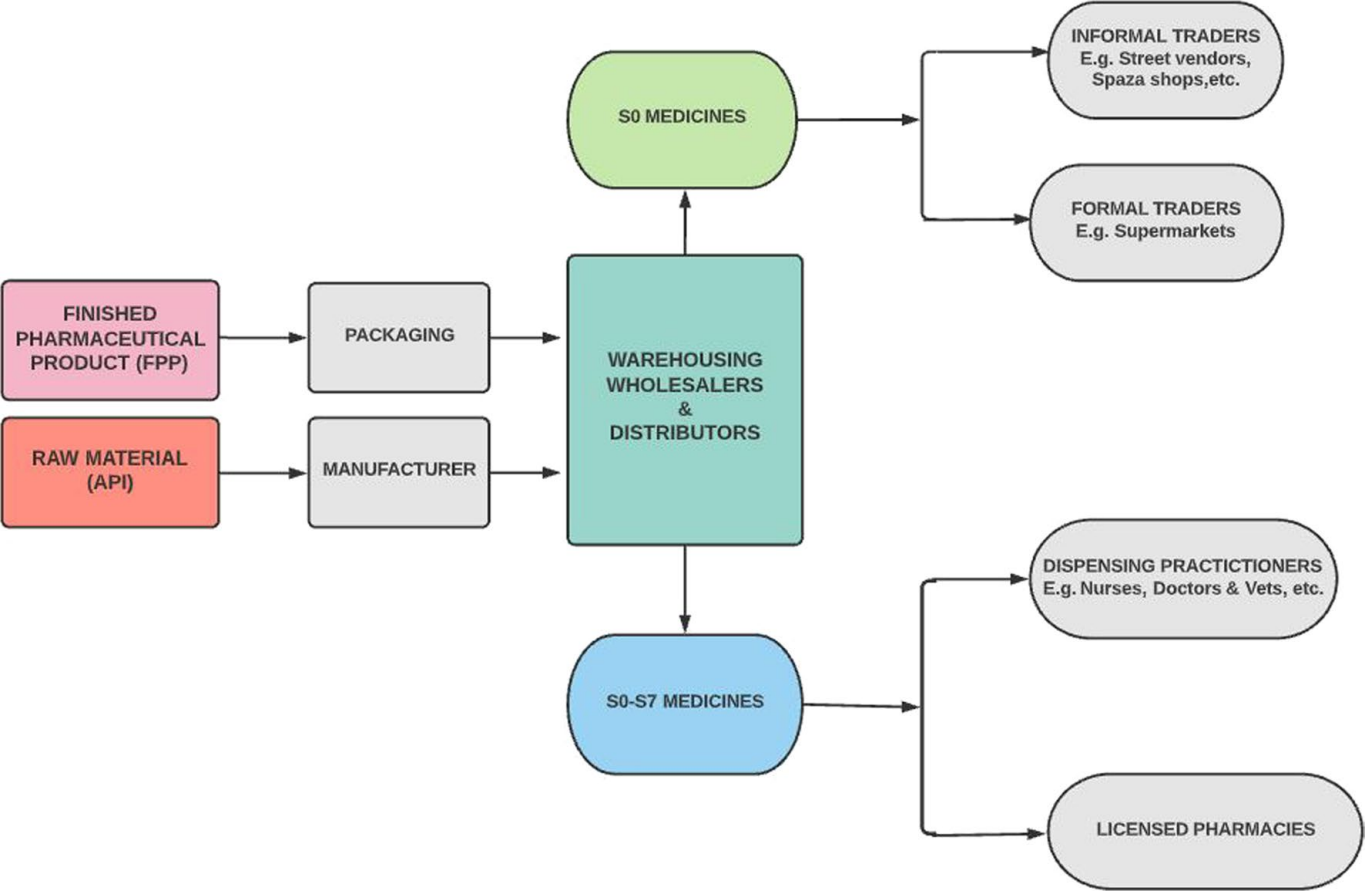
South African medicines supply chain showing the route a Finished Pharmaceutical Product (FPP) and an Active Pharmaceutical (API) takes to reach the end user (consumer or patient)

**Fig. 3:**
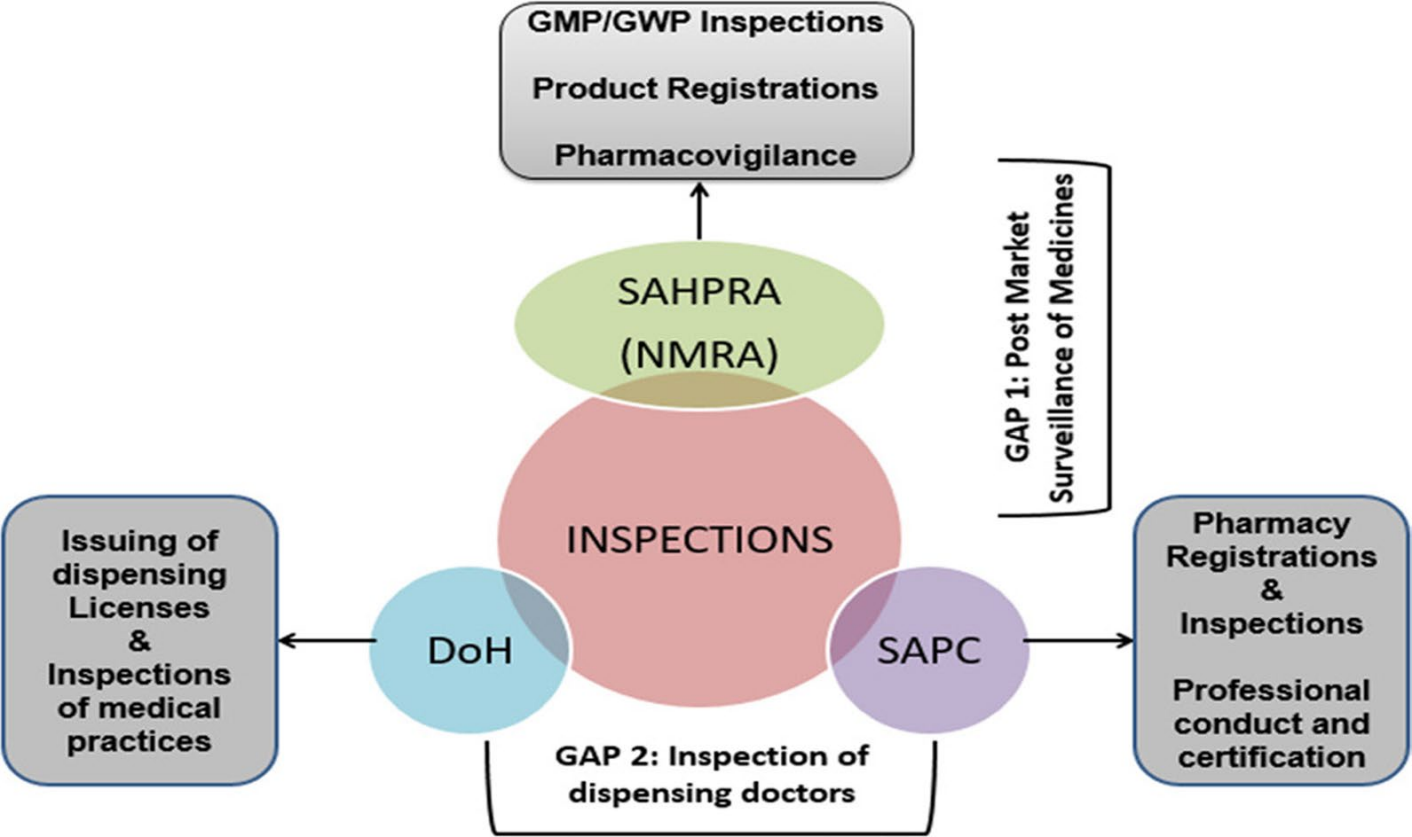
Two gaps in the shared inspection function of government agencies dealing with the regulation of medicines in South Africa. Gap 1: Exists in lack of Post Market Surveillance once products have been registered due to restrictions in jurisdiction. Gap 2: Exist in the case of inspecting dispensing doctor or medical practitioners. The Department of Health (DoH) was unable to follow through due to limited capacity, while the South African Pharmacy Council (SAPC) couldn’t step into the role due to jurisdiction as well

The interviews show that South Africa has a good regulatory policy and legislative framework; however, implementation remains a challenge as far as SFs and post market surveillance is concerned [35]. Consequently, there need arises for a more specific legislation to address SFs with a clear implementation strategy. There are visible gaps in the supply chain making infiltration possible especially with the informal markets that are allowed to sell schedule 0 medicines, such as some pain medication, for example, 500 mg paracetamol (see Fig. 2). The second gap exists in inspections (see Fig. 3), relating to private medical practitioners with dispensing licences which are currently not inspected as they should primarily due to limited human resources and conflicting mandates between government health agencies.

Our study found that, the regulation of pharmaceuticals in South Africa is governed by the Medicines and Related Substances Act of 101 of 1965 (101/65) and is implemented by the NMRA, the South African Health Products Regulatory Authority also known as the former Medicines Control Council (MCC). The enforcement of the law, however, involves other stakeholders and the use of other pieces of legislation. The lack of harmonisation between the Medicines and Related Substances Act 101 of 1965 and the Counterfeit Goods Act 37 of 1997 and other legislation such as the Prevention of Organised Crime Act 121 of 1998 and the Electronics and Communications Act 25 of 2002, opens up gaps in the regulatory and criminal justice system giving criminals loopholes to manipulate court proceedings and prosecutions. The focus of the law in general was found to be on false advertising, labelling, trademark infringement and operation of illegal pharmacy premises and not necessarily illegal online pharmacies or counterfeit and substandard medicines (see Table 6). Penal Sanctions were also found to be weak, with only a manageable business cost charged and a light prison sentence. The outcome of prosecutions was also poor due to the complexities associated with court proceedings and short timelines. This outcome was also due to the lack of political and technical expertise on how to handle pharmaceutical crimes within the criminal justice system. Office on Drugs and Crime’s research brief [36].

**Table 6:** Three identified companies trading in online unauthorised pharmaceutical products on the South African market

Name of websites	Type of product	Website address	Country of origin
Monarch medical supplies	Illegal supply of registered and unregistered medicines Controlled medicines sold without prescription	http://monarchmedsupply.com	Contact details form Switzerland
Direct pharma supplies	Medical devices	info@directdermasupplies.com	Contact details from Denmark
Medicair	Illegal supply of registered and unregistered medicinesControlled medicines sold without prescription	www.medicair.co.za	South Africa Note: Contact details were US and UK phone numbers instead of SA

In response to this problem, Kenya enacted the anti-counterfeiting Act 13 of 2008 [37] which was later revised to facilitate access to generic medicines for HIV/AIDS. The lack of harmonisation between the aforementioned Act and the already existing pieces of legislation—the Industrial Property Act of 2001 and the HIV Prevention Control Act of 2006 resulted in generic medicines being categorised as counterfeit which made the revision necessary.

It is considered best practice for an anti-counterfeiting policy framework to encompass the following key components: product and supply chain security, advocacy, engagement and awareness and risk-based enforcement and threat assessment [38]. Specific policy objectives should include the prevention of manufacturing and distribution of SFs (including APIs), securing the legal distribution chain against SFs prohibiting importation of SFs and participating in the regional and global campaigns [39]. Such a policy should also entail restrictions on the trade of pharmaceuticals online and the legal requirements for operating online pharmacies.

### Institutional resources

The limited availability of inspectors in the NMRA assigned to perform inspections and post market surveillance was cited as a great concern. The COVID-19 pandemic has placed an even greater demand on movement of health related products across borders. This has in turn slowed down inspections and visibility of officers especially due to the emphasis on ‘free trade zones’ in the region. This provides a clear gap for cross border criminal activity. Research has shown that infiltration of counterfeit medicines is highest in places, where law enforcement is weakest [40]. Increased visibility of inspectors at ports of entry and cross skill training of regulatory will have a huge impact on curbing transnational crimes of this nature.

Funding plays an important role in facilitating enforcement work and developing countries such as South Africa can benefit from collaborative efforts of international agencies, such as the joint trilateral relationship between the WHO, UNODC and Interpol [18]. A multi-pronged approach is required for effective combat, hence each of these parties has a unique role to play within its own mandate without infringing on the other. The debates between public health and intellectual property infringements has hindered efforts in the past leading to the collapse of agencies, such as the WHO IMPACT in 2010 [41].

### Pharmaceutical testing facilities

Developed countries have more advanced laboratory infrastructure and technology. For example, in the EU, the European Directorate for Quality Medicines (EDQM) has developed a fingerprint database of active ingredients used in the manufacture of medicines which can be identified by analytical methods, such as chromatography and spectrophotometry [42, 43]. The information generated by the analytical process can be used by regulators to provide evidence and, therefore, pursue appropriate legal action. It is evident that partnerships between international stakeholders make in-country capacity building possible in resource-limited nations [44].

Such can be seen in the case of the partnership between the Global Pharma Health Fund (GPHF) and the Centre for Pharmaceutical Advancement and Training (CePAT), and the United States Pharmacopeia and Ghana [45]. Similarly, Nigeria has had great success by having the ISO/IEC 17025 accreditation of its five public sector medicines testing laboratories through the on-going support of the U.S Pharmacopeial Convention (USP), [46].

The purpose of such collaborations is to build in-country and regional capacity in pharmaceutical quality assurance and quality control; however, the onus is with individual countries to take ownership of the process. Collaborations and partnerships like these can be equally beneficial to South Africa as it tries to address its own capacity building needs.

### Stakeholder collaboration and information sharing

Over the past decade, the international community has made significant efforts to improve cooperation and collaboration among national law enforcement agencies involved in medical-related crime. Unfortunately, the lack of coordination amongst public agencies undermines these efforts. This remains a challenge in most developing countries as is the case with South Africa. These gaps in enforcement have become more evident during the COVID-19 global crisis [47].

A more transparent relationship with all parties involved should be encouraged by the South African NMRA by sharing information with respect to registered products and counterfeit incidents. For example, by providing real-time visibility to products manufactured and exported such as in the case of India (India Department of Commerce [48]). The Indian Drug Authentication and Verification Application (DAVA) programme has empowered consumers to be able to check barcodes of medicines brought against an existing database on the website. Furthermore, funding should be made available for health promotion campaigns so that the public can be educated.

It is, therefore, necessary to introduce a policy that will mandate stakeholder engagement, collaboration, and information sharing. Such should also promote the fostering of industry participation through public–private partnerships. For example, private partners in other countries, such as United States and the European Union include: The Pharmaceutical Security Institute (PSI), Quality Brands Protection Committee of China (QBPC), International Federation of Pharmaceutical Manufacturers Association (IFPMA)—*“Fight the fake”* partnerships and the International Anti-Counterfeiting Coalition (IACC) [49].

Furthermore, the use of a central database can be used to synchronise the regional policing of the SFs. Reports of threat-based assessments should be used to guide investigations and inspections [50]. For instance, based on annual report analysis for the financial year [51], the agency has already implemented a regional with SADC some member States electronic information sharing system in partnership with the World Customs Organisation (WCO) and the Organisation for Economic Co-operation and Development (OECD). Towards enabling risk-assessments at ports of entry, SADC members such as Mozambique, Swaziland, Mozambique, and Malawi are cooperating with South Africa and the WCO. Unfortunately, there is no information regarding whether medicines are included in what is considered high risk.

### Enforcement and post market surveillance

The use of forensic intelligence techniques such as DNA fingerprinting and incident trend analysis will greatly enhance law enforcement efforts. Investment in detection tools such as the German Pharma Fund portable minilabs which use Raman spectroscopy and Near Infrared (NIR) spectroscopy and the *Truscan* handheld device [52] to minimise the long waiting periods for laboratory results and improved information sharing of results in a database such as the WHO’s *Vigibase* system will help support any legal proceedings instituted [53].

## Conclusion

From both the document analysis and the interviews findings, South Africa has an existing National Drug Policy and legislative framework that is compatible to international standards [54]. However, there is no existing national anti-counterfeiting strategy to address medicines. The implication of the absence of such a policy are clear. It can be seen in the lack of coordination between governmental departments, lack of stakeholder engagement and collaboration (private–public partnerships), lack of harmonisation on existing legislation, lack of capacity and resource mobilisation subsequently hampering effective law enforcement.

Another point highlighted by the study was the poor outcome in prosecutions of cases. This was cited to have been greatly influenced by the lack of specific pharmaceutical legislation providing guidelines on how to address counterfeit medicines and the lack of harmonisation of existing laws. Although the existing legislation is effective in addressing issues of IP infringement such as the Counterfeit Goods Act (trademark and copyright infringement) and Prevention of Organised Crime Act (asset forfeiture) in most cases prosecutors were said to not be knowledgeable of handling pharmaceutical-related crime. The sanctions were also not deterrent enough.

Again, the penal sanctions stipulated in the Medicines and Related Substances Act were also found to not be specific to counterfeit medicines and were not deterrent enough. Successful cases in our study are due to administering a combination of all three pieces of legislation together. It is, therefore, worth considering the harmonisation of the existing legislation to effectively combat SFs and to implement an anti-counterfeiting policy.

The study also illuminated that although law enforcement agencies had various combat activities to varying degrees around counterfeit merchandise, pharmaceutical crime was not the focus. This lack of coordination of enforcement activities opens up gaps in the regulation chain for the infiltration of SFs. The NMRA was also seen to have taken a more passive role in leading the fight and some institutions cited a lack of coordination to be due to jurisdictional limitations and competing mandates. Recognition of the problem at hand and active engagement of key stakeholders is crucial in combating pharmaceutical crime at national and regional levels.

## Practical implications and future research

The findings of this study should be considered as an exploratory analysis that will generate a hypothesis for further investigation on policy interventions to combat SFs.

### Developing an anti-counterfeit policy and strategy

There is a need for a specific pharmaceutical anti-counterfeit policy that can address the unique complexities involved such as the IPR issues, safety and efficacy of medicines and the legal implications thereof that the current legislation does not effectively cater for. The implementation of the national anti-counterfeit policy will enforce a legal mandate with objectives and responsibilities for the effective participation of all stakeholders. Amendments that will include an anti-counterfeiting plan and clear objectives for establishing a Post Market Surveillance and a Pharmacovigilance plan can be valuable. This is considered best practice [55].

#### Harmonisation of existing laws

In retrospect, the alignment of existing legislation to employ a more integrated approach between stakeholders is an important consideration and should be mandated in the aforementioned policy. A thorough process of impact assessment and feasibility studies should be undertaken to ensure the best policy options that suit the South African context. It is considered best practice for an anti-counterfeiting policy framework to encompass the following key components: supply chain security at company, national and regional levels, stakeholder engagement and awareness as well as threat assessment and risk-based enforcement strategies [56, 57].

#### Improving visibility of anti-counterfeit campaigns

The national regulator, SAHPRA should lead the fight against SFs and engage all stakeholders including the public. With the growing risks of internet pharmaceutical crime, educating the public on how to identify SFs and on safe online buying of medicines is equally of value and using readily accessible media platforms such as newspapers, radio, television and social media can be effective [58].

#### Strengthening stakeholder collaboration

In addition, strengthening existing inter-agency relationships and regional collaborations is key. The study has shown that due to the weak stakeholder relationships collaborations are not always possible. As a result, duplicity of shared enforcement function often can be seen with little impact on the SFs. These can be converted into synergistic efforts drawing on the strength in resources and capacity of all players.

#### Accurate data reporting and risk based assessment

Finally, the study has shown that there is very little reporting on SFs and poor data collection making it difficult to measure the true extent of the SF problem in South Africa. Proper data capturing and reporting provides important information to policy makers and makes it easier to advocate for deployment of the necessary funding and resources to achieve the desired outcome.

## Supplementary Information

**Additional file 1.** (addendum A): Informed consent.

**Additional file 2**. (addendum B): Interview guide.

**Additional file 3.** (addendum C): request letter for interviews.

**Additional file 4.** (addendum D): ethical approval letter.

**Additional file 5.** (addendum E): data collection tool for document analysis.

**Additional file 6.** (addendum F): list of websites and databases.

**Additional file 7.** (addendum G): SA legislative framework.

**Additional file 8.** (addendum H): Criteria for trustworthiness in the research process.

## Data Availability

The data sets generated during and/or analysed during the study are available from the corresponding author on reasonable request.

## Abbreviations

ADRs: Adverse Drug Reactions
AIDAN: All India Drug Action Network
AIDS: Acquired Immune Deficiency Syndrome
API: Active Pharmaceutical Ingredient
CIPC: Companies intellectual Property Commission
COE: Council of Europe
COVID-19: Corona virus disease 2019
DAVA: Drug Authentication and Verification Application
DHA: Department of Home Affairs
DoH: Department of Health
DRC: Democratic Republic of Congo
DTI: Department of Trade and Industry
DNA: Deoxyribonucleic acid
EAC: East African Community
EQDM: European Directorate for Quality of Medicines and healthcare
EUROPOL: European Law Enforcement Agency
FDA: Food and Drug Administration (US)
FIP: International Pharmaceutical Federation
GMP: Good Manufacturing Practice
GTIN: Global Trade Item Number
HIV: Human Immuno-deficiency Virus
IACC: International Anti-Counterfeiting Coalition
ICH: International Council for Harmonisation
IFPMA: International Federation of Pharmaceutical Manufacturers Association
INTERPOL: International Police Organisation
IP: Intellectual Property
IPR: Intellectual Property Rights
IRACM: International Institute of Research against Counterfeit Medicines
MCC: Medicines Control Council
MHRA UK: United Kingdom Medicines and Healthcare Products Regulatory Agency
MRSA: Medicines and Related Substances Act 101 of 1965
NAFDAC: National Agency for Food and Drug Administration and Control (Nigeria)
NDP: National Drug Policy
NIR: Near Infra-red
NDP: National Drug Policy
NMRA: National Medicines Regulatory Authority
NPA: National Prosecuting Authority
OECD: Organisation for Economic Cooperation and Development
PMS: Post Market Surveillance
PSI: Pharmaceutical Security institute
QBPC: Quality Brands Protection Committee of China
SA: South Africa
SADC: Southern African Development Community
SAPC: South African Pharmacy Council
SAHPRA: South African Health Products Regulatory Authority
SAPS: South African Police Services
SARS: South African Revenue Services
SF: Substandard and Falsified
SSFFC: Substandard/Spurious/Falsely Labelled/Fake/Counterfeit
TB: Tuberculosis
TRIPs: Trade Related Intellectual Property Agreements
UN: United Nations
UNODC: United Nations Office for Medicine Control and Crime prevention
USAID: United States Agency for International Development
VIPPS: Verified Internet Pharmacy Practice Sites (VIPPS)
WCO: World Customs Organization
WHA: World Health Assembly
WHO: World Health Organization
WIPOTEC-OCS: An acronym meaning Outstanding Manufacturing Quality “Made in Germany”
