# Supplementary material for: An exploratory assessment of the legislative framework for combating counterfeit medicines in South Africa
Source: J Pharm Policy Pract. 2022 Jan 5;15:3. doi: 10.1186/s40545-021-00387-8 (PMC8730303; doi:10.1186/s40545-021-00387-8)
Supplement: Supplementary file 1 — Additional file 1. (addendum A): Informed consent. [file 40545_2021_387_MOESM1_ESM.docx]

# \

# ADDENDUM A


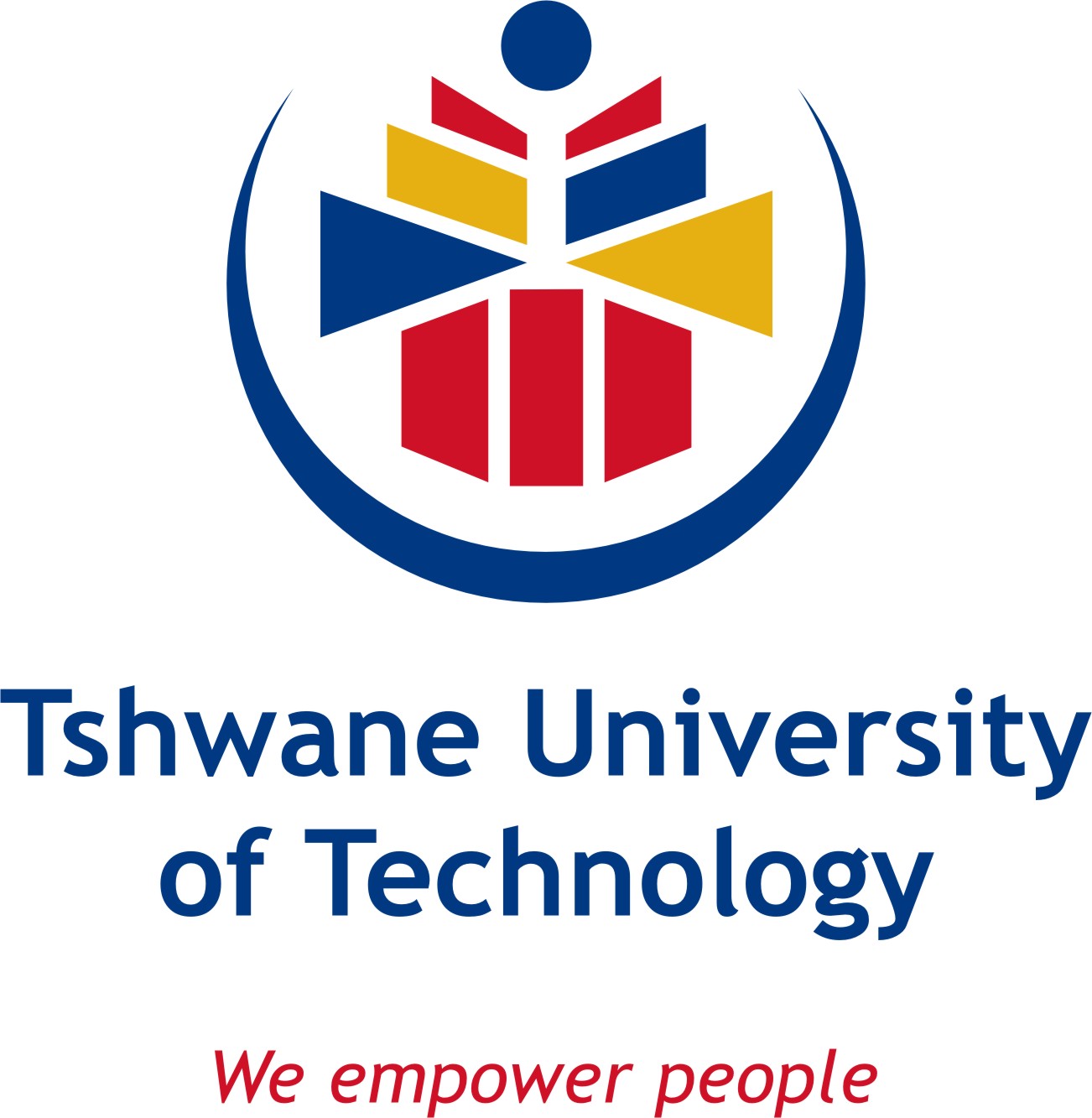


### FACULTY OF SCIENCE

DEPARTMENT OF PHARMACEUTICAL SCIENCES

####

#### PARTICIPANT INFORMATION LEAFLET AND INFORMED CONSENT

**PROJECT TITLE:**

**ASSESSING THE LEGISLATIVE AND POLICY FRAMEWORK FOR COMBATING COUNTERFEIT MEDICINES IN SOUTH AFRICA**

### Dear Participant,

You are invited to be interviewed on the above mentioned subject as part of the formal Masters studies. This information leaflet will help you understand more about the study and hence enable you to make an informed decision to participate or not. Before, agreeing to participate please make sure that you understand and are comfortable with what will be required of you should you be interested. You are at liberty to decline to participate if there are aspects of the study you do not agree to.

**WHAT IS THE STUDY ABOUT?**

The study will focus on the issue of counterfeit medicines with a special interest in the South African situation. According to the World Health Organization (WHO) SFs which is an acronym for Substandard and Falsely labelled medical products is defined as, “medicines which are deliberately and fraudulently mislabelled with respect to identity and/or source.” Both originator and generic medicines can be counterfeited in various ways such as inclusion of wrong ingredients, absence of active pharmaceutical ingredients, inconsistent formulations or fake packaging. Counterfeit medicines are found in both industrialised and developing countries and they generally follow the dominant drug use patterns. In developing countries the counterfeiters’ main targets are medicines for infectious diseases such as Malaria, Tuberculosis and Human Immunodeficiency Virus and Acquired Immunodeficiency Syndrome (HIV and AIDS). In industrialized countries the targets tend to be lifestyle medicines such as anti-depressants or treatments for erectile dysfunction, hair loss, weight management and most recently cancer treatment. Counterfeiting of pharmaceutical drugs has an adverse impact at the individual level, within the health care system as well as for pharmaceutical companies and economies of countries. Of particular interest to this study is public health safety. Counterfeit drugs can lead to deaths and drug resistant-treatment failure for serious life threatening conditions like HIV/AIDS and Tuberculosis.

**The aims of the study are to:**

To assess the legislative and policy framework and institutional arrangements which govern pharmaceuticals and the anti-counterfeiting strategies therein and to evaluate the level of awareness and stakeholder collaboration.

**WHAT WILL YOU ARE EXPECTED TO DO IN THE STUDY?**

If you decide to take part in the study, you will be required to sign an informed consent form of which you will be given a copy by the researcher.

You will have to answer open ended questions in a face to face or telephonic interview. You will be asked to respond to questions regarding incidents of counterfeit medicinal products, processes of handling counterfeit products, routine quality checks and annual records/reports on seized counterfeit products as well as combat initiatives and collaborations aimed at raising awareness. It should not take more than 1 hour per session for interviews and not more than 45 minutes to complete questionnaires. Follow up telephonic interviews will take between 15 and 20 minutes if required.

**WHAT CONDITIONS MAY EXCLUDE YOU FROM THE STUDY?**

The study is targeted at persons and organizations involved in the medicines supply chain and law enforcement in South Africa but not at individual consumers or persons below 18 years of age.

**ARE THERE ANY FORESEEABLE RISKS TO PARTICIPATING IN STUDY?**

The interviews involve no foreseeable threat to your career or position in your current work environment. The questions asked will not be of a personal nature and will be used for information purposes about the topic of the study.

During interviews you will be requested to share information on agency records on ensuring quality of operations (Standard Operating Procedures) in handling confiscated counterfeit medicinal products and information regarding involvement of the organisation with other stakeholders. This information will be treated with strict confidentiality to avoid the loss of any trade advantage or reputation.

**WHAT ARE THE POTENTIAL BENEFITS THAT MAY COME FROM THE STUDY**

The results of the interviews will have no direct personal benefit to you, but you will make a meaningful contribution towards a better understanding of the nature of counterfeit medicines, the issues around combat and the impact in South Africa. There will also be a mutual benefit of information sharing amongst key stakeholders in the pharmaceutical supply chain to better inform and strengthen existing policies and awareness efforts.

**WILL YOU RECEIVE ANY FINANCIAL COMPENSATION OR INCENTIVE FOR PARTICIPATING IN THE STUDY?**

Please note that you **will not** be paid to participate in the study

**WHAT ARE YOUR RIGHTS AS A PARTICIPANT IN THE STUDY?**

Your participation in this study is entirely voluntary and anonymous. You have the right to withdraw at any stage without any penalty or future disadvantage whatsoever. You don’t even have to provide the reason/s for your decision. Your withdrawal will in no way influence your continued relationship with the research team. Note that you are not waiving any legal claims, rights or remedies because of your participation in this research study. All information obtained from the questionnaire is strictly confidential.

**HOW WILL CONFIDENTIALITY AND ANONYMITY BE ENSURED IN THE STUDY?**

The interview data will remain confidential and your identity will not be revealed while the study is being conducted or when the study is reported in scientific journals and/or research reports. All the hard copies of interview transcripts will be stored in a secure place at the Tshwane University of Technology for three years, after which they will be destroyed. Any information that is obtained in connection with this study and that can be identified with you will remain confidential and will be disclosed only with your permission or as required by law. The information received during the project will only be used for research purposes and not be released for any employment-related performance evaluation, promotion and/or disciplinary purposes. Access to your data will be strictly limited to the researcher, the supervisors of the study and the designated examiners (appointed by Tshwane University of Technology).

**IS THE RESEARCHER QUALIFIED TO CARRY OUT THE STUDY?**

Yes. The researcher holds a B-Tech in Pharmaceutical Sciences and has done research projects before. She has also has had further training by attending the Research Methodology course that is offered by the Tshwane University of Technology annually.

**HAS THE STUDY RECEIVED ETHICAL APPROVAL?**

The study has gone through a thorough ethics review process has been granted approval by the Faculty Committee for Postgraduate Studies and the Research Ethics Committee of the Tshwane University of Technology. All parts of the study will be conducted according to internationally accepted ethical principles.

**WHO CAN YOU CONTACT FOR ADDITIONAL INFORMATION ABOUT THE STUDY?**

#### The primary investigator and the study leader can be contact during office hours by telephone or by email. Should you have any questions regarding the ethical aspects of the study, you can contact the chairperson of the TUT Research Ethics Committee. Alternatively, you can report any serious unethical behaviour at the University’s Toll Free Hotline 0800 21 23 41.

**DECLARATION: CONFLICT OF INTEREST**

None of the companies or agencies participating has any financial gains to be made from or have contributions towards the study or to the Tshwane University of Technology. This research study was funded by the Tshwane University of Technology. No publication prohibitions, conditions or limitations were placed on the researcher.

**FINAL WORD**

Thank you for taking the time to read through the information leaflet. Your participation in the study will be greatly appreciated. Please sign the informed consent below if you agree to participate in the study.

**CONSENT**

I hereby confirm that I have been adequately informed by the researcher about the nature, conduct, benefits and risks of the study. I have also received, read and understood the above written information. I am aware that the results of the study will be anonymously processed into a research report. I understand that my participation is voluntary and that I may, at any stage, without prejudice, withdraw my consent and participation in the study. I had sufficient opportunity to ask questions and of my own free will declare myself prepared to participate in the study.

Research participant’s name: (Please print)

Research participant’s signature:

Date:

Researcher’s name: (Please print)

Researcher’s signature:

Date:
