# Supplementary material for: An exploratory assessment of the legislative framework for combating counterfeit medicines in South Africa
Source: J Pharm Policy Pract. 2022 Jan 5;15:3. doi: 10.1186/s40545-021-00387-8 (PMC8730303; doi:10.1186/s40545-021-00387-8)
Supplement: Supplementary file 2 — Additional file 2. (addendum B): Interview guide. [file 40545_2021_387_MOESM2_ESM.docx]

# ADDENDUM A


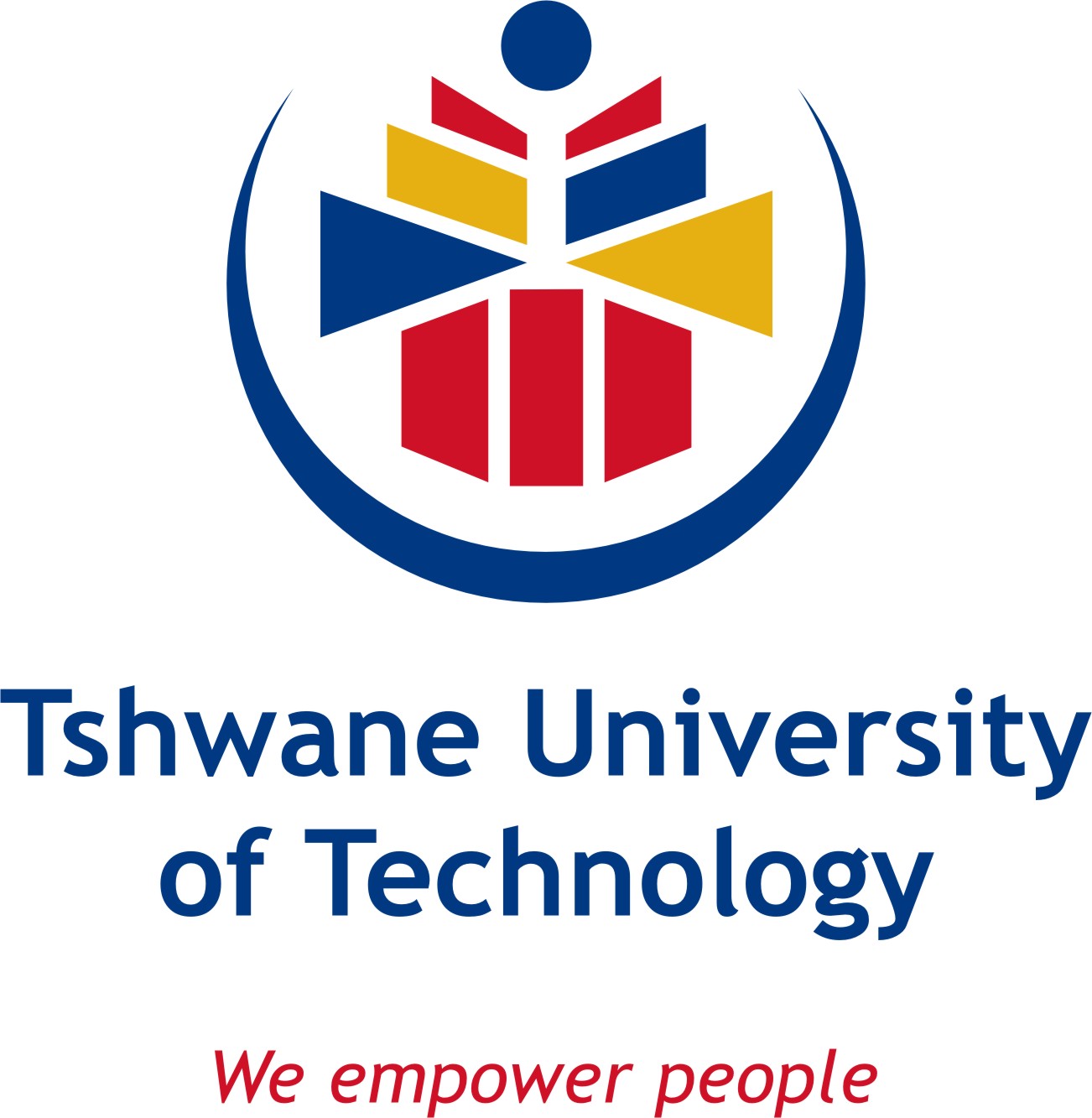


### FACULTY OF SCIENCE

DEPARTMENT OF PHARMACEUTICAL SCIENCES

**I**

| **INTERVIEW GUIDE** |
| --- |
| **Legislation** |
| What kind of legislation is used to in South Africa to combat counterfeit medicines (SF)? 2) Does it adequately address the issue of SF? 3) Does the legislation define SF? 4) What areas need revision? 5) How does the legislation penalize criminals of SF? 6) Is the punishment adequate? 7) Are there designated ports of entry for importation of pharmaceuticals? |
| **Limited resources** |
| How many inspectors or officials conduct inspections? 2) Give the number of seizures of SF between the past two years? 3) What was the source of origin of these SF? 4) What authorities handled the case? 5) Was any laboratory testing done on samples? If not, what was the reason? 6) Are there any designated or accredited laboratories authorised to test samples of seized products by the NMRA? 7) How long does it take to results back? 8) Of the confirmed cases how many have been successful prosecuted? 9) Are there any training initiatives in place on SF? |
| **Enforcement** |
| What regulatory measures are being used to combat SF in the country? 2) Which products have been counterfeited (therapeutic class e.g. analgesics, anti-diabetic etc.) the most and from which country of origin? 3) Is there a protocol for assessing seized SF products, to manage records, sampling, destroying, and communication on confirmed cases? 4) How is conflict of interest managed in the department/agency? |
| **Market Control** |
| Are all distribution channels authorized? 2) What is the estimated number of illegal outlets? 3) What is the percentage distribution of medicines among different stakeholders (public, private, informal, NGOs)? 4) Are there a significant proportion of medicines on the market without proper marketing authorisation in South Africa? 5) How many samples have been collected and tested for purposes of detecting counterfeits? 6) Are companies encouraged to have anti-counterfeit security features on their products? If yes, how is the public informed about them? |
| **Stakeholder collaboration: National, Regional and International & Information Sharing** |
| Is there an anti-counterfeiting task team in the department or agency? 2) describe any collaboration with different stakeholders locally and regionally? 3) Are there any joint anti-counterfeiting operations with other international agencies? 4) Is it mandatory to inform the NMRA? 5) Give the number of cases you informed at least one country? |
| **Education and Awareness** |
| Are there any national awareness campaigns you know of locally? 2) Who is the target audience? 3) Are confirmed cases made public? 4) How is information disseminated? |
