# Supplementary material for: An exploratory assessment of the legislative framework for combating counterfeit medicines in South Africa
Source: J Pharm Policy Pract. 2022 Jan 5;15:3. doi: 10.1186/s40545-021-00387-8 (PMC8730303; doi:10.1186/s40545-021-00387-8)
Supplement: Supplementary file 3 — Additional file 3. (addendum C): request letter for interviews. [file 40545_2021_387_MOESM3_ESM.docx]

# ADDENDUM A


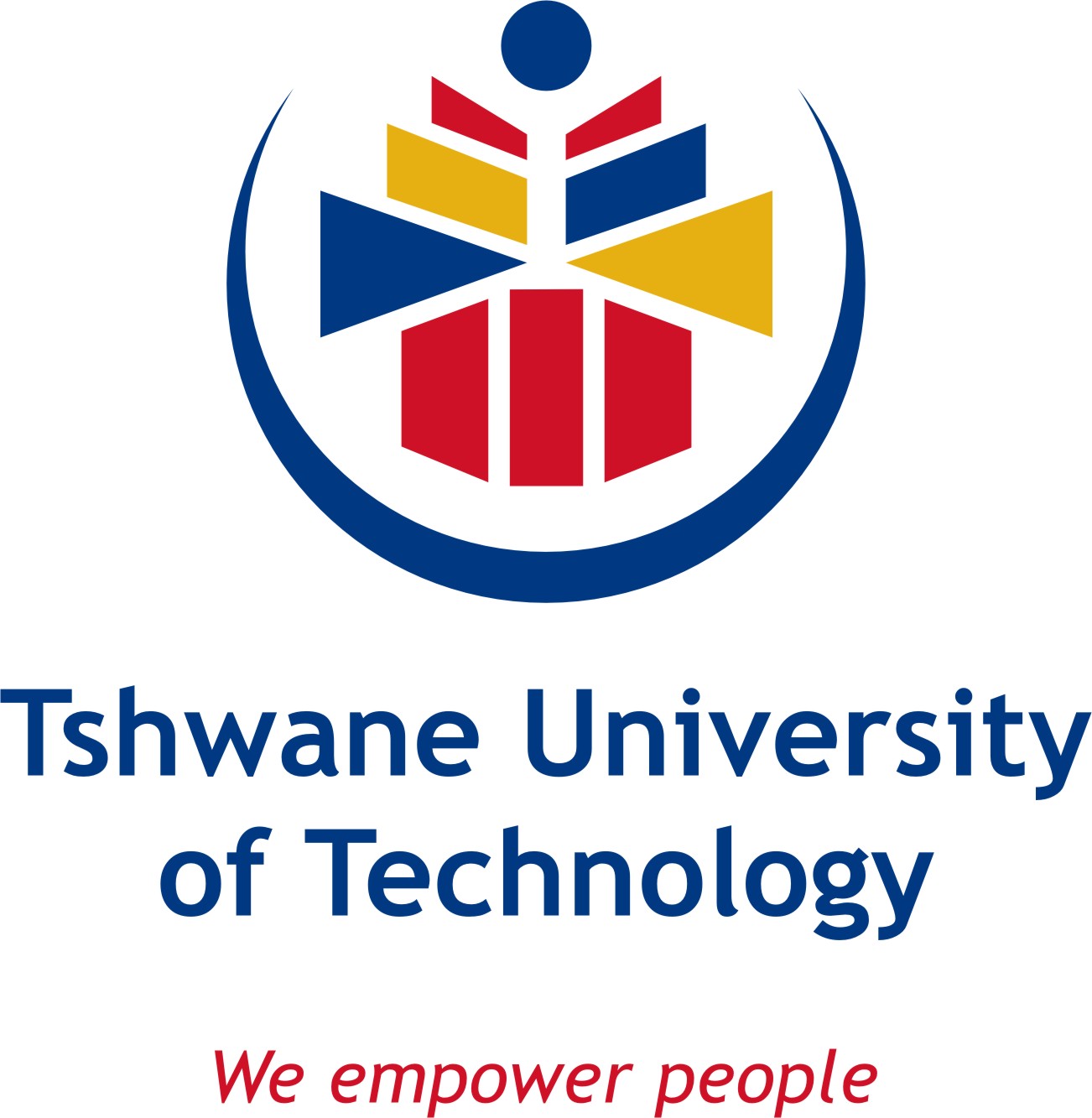


### FACULTY OF SCIENCE

DEPARTMENT OF PHARMACEUTICAL SCIENCES

#### COVER LETTER – REQUEST FOR INTERVIEWS

**ASSESSING THE LEGISLATIVE AND POLICY FRAMEWORK FOR COMBATING COUNTERFEIT MEDICINES IN SOUTH AFRICA**

### Dear Potential Research Participant,

You are invited to complete a survey questionnaire and to be interviewed on the above mentioned subject as part of my formal Masters studies.

1. BACKGROUND AND JUSTIFICATION

**Introduction**

According to the World Health Organization (WHO), ‘Substandard and Falsified (SF) medical products are medicines which are deliberately and fraudulently mislabelled with respect to identity and/or source. ‘Counterfeit’ has a specific meaning in intellectual property that is related to wilful trademark violations. In relation to medicines it is used in a much broader sense to do with misrepresentation of identity or source, or even medicines that are simply ‘substandard’. Some countries use the term ‘falsified’ to describe medicines that misrepresent their identity or source, but do not necessarily violate intellectual property rights. ‘Substandard’ medicines are those that do not meet quality standards specified for them, but may also be defined specifically to cover products from authorized manufacturers which fail to meet quality standards set for them. Both originator and generic medicines can be counterfeited in various ways such as inclusion of wrong ingredients, absence of active pharmaceutical ingredients, inconsistent formulations or fake packaging. Counterfeit medicines are found in both industrialised and developing countries and they generally follow the dominant drug use patterns; that is in developing countries the counterfeiters’ main targets are medicines for infectious diseases such as Malaria, Tuberculosis and Human Immunodeficiency Virus and Acquired Immunodeficiency Syndrome (HIV and AIDS). In industrialized countries the targets tend to be lifestyle medicines such as anti-depressants or treatments for erectile dysfunction, hair loss, weight management and most recently cancer treatment. Over the past decade, there has been a growing concern globally with the increase in the manufacturing and trade of counterfeit medicines. Counterfeiting of medicines has a negative impact on global efforts to treat life threatening diseases such as mentioned previously by facilitating drug resistance. It also negatively affects the economies of countries as well as individual pharmaceutical company revenues.

**Aims and objectives**

To assess the legislative and policy framework and institutional arrangements which govern pharmaceuticals and the anti-counterfeiting strategies therein and to evaluate the level of awareness and stakeholder collaboration.

**EXPECTATIONS ON PARTICIPATION**

If you decide to take part in the study, you will be required to complete face to face, one on one or telephonic interview. You will be asked to respond to questions regarding incidents of counterfeit medicinal products, processes of handling confiscated products and annual records/reports on seized counterfeit products as well as combat initiatives and collaborations on raising awareness. It should not take more than 45 minutes to complete it.

**EXCLUSION CRITERIA**

Individual consumers, retailers (pharmacy/dispensers) the medicine supply chain in South Africa or persons below the age of 18 years.

**POTENTIAL AND/OR FORESEEABLE RISKS**

Participation in the interview involves no foreseeable threat to your career or position in your current work environment. The questions asked will not be of a personal nature and will be used for information purposes around the topic of the study. In the interviews you will be requested to information on quality assurance, Standard Operating Procedures (SOP) and confiscated counterfeit medicinal products and information regarding your company / business. This information will be treated with strict confidentiality to avoid the loss of any trade advantage by your company / business.

**POTENTIAL BENEFITS**

The results of the interview will have no direct personal benefit to you, but you will make a contribution towards a better understanding about the nature of counterfeit medicines, their impact on public health and on the economy of the country. As well as the mutual benefit of information sharing amongst key stakeholders in the pharmaceutical supply chain to better inform and strengthen existing policies and implementation of strategies in the fight against counterfeiting of medicines.

**BASIC RIGHTS OF THE PARTICIPANT**

Your participation in this study is entirely voluntary and anonymous. You have the right to withdraw at any stage without any penalty or future disadvantage whatsoever. You don’t even have to provide the reason/s for your decision. Your withdrawal will in no way influence your continued relationship with the research team. Note that you are not waiving any legal claims, rights or remedies because of your participation in this research study. All information obtained from the questionnaire is strictly confidential.

The interview data and demographic information will be coded so that it will not be linked to your name. Your identity will not be revealed while the study is being conducted or when the study is reported in scientific journals and/or research reports. All the hard copies of the questionnaires that have been completed will be stored in a secure place at the Tshwane University of Technology for three years, after which they will be destroyed. Any information that is obtained in connection with this study and that can be identified with you will remain confidential and will be disclosed only with your permission or as required by law. The information received during the project will only be used for research purposes and not be released for any employment-related performance evaluation, promotion and/or disciplinary purposes. Access to your data will be strictly limited to the researcher, the supervisors of the study and the designated examiners (appointed by Tshwane University of Technology).

**ETHICAL CONSIDERATIONS**

The study has undergone a thorough ethics review process and has been approved by the Faculty Committee for Postgraduate Studies and the Research Ethics Committee of the Tshwane University of Technology. Also, the management of National Medicines Regulator, the Medicines Control Council and both identified wholesalers and manufacturers of medicinal products have granted written consent and approval for the study. All parts of the study will be conducted according to internationally accepted ethical principles.

Contact Information for the Researcher, Study Supervisor(s) and Ethics Committee.

The primary investigator and the study leader can be contact during office hours by telephone or by email. Should you have any questions regarding the ethical aspects of the study, you can contact the chairperson of the TUT Research Ethics Committee. Alternatively, you can report any serious unethical behaviour at the University’s Toll Free Hotline 0800 21 23 41.

**CONFLICT OF INTEREST**

None of the companies or agencies participating has any financial gains to be made from or have made contributions towards the study or the Tshwane University of Technology.

Your participation in the study will be greatly appreciated.
