# Supplementary material for: An exploratory assessment of the legislative framework for combating counterfeit medicines in South Africa
Source: J Pharm Policy Pract. 2022 Jan 5;15:3. doi: 10.1186/s40545-021-00387-8 (PMC8730303; doi:10.1186/s40545-021-00387-8)
Supplement: Supplementary file 4 — Additional file 4. (addendum D): ethical approval letter. [file 40545_2021_387_MOESM4_ESM.docx]

# ,m

### FACULTY OF SCIENCE

# ADDENDUM A

DEPARTMENT OF PHARMACEUTICAL SCIENCES


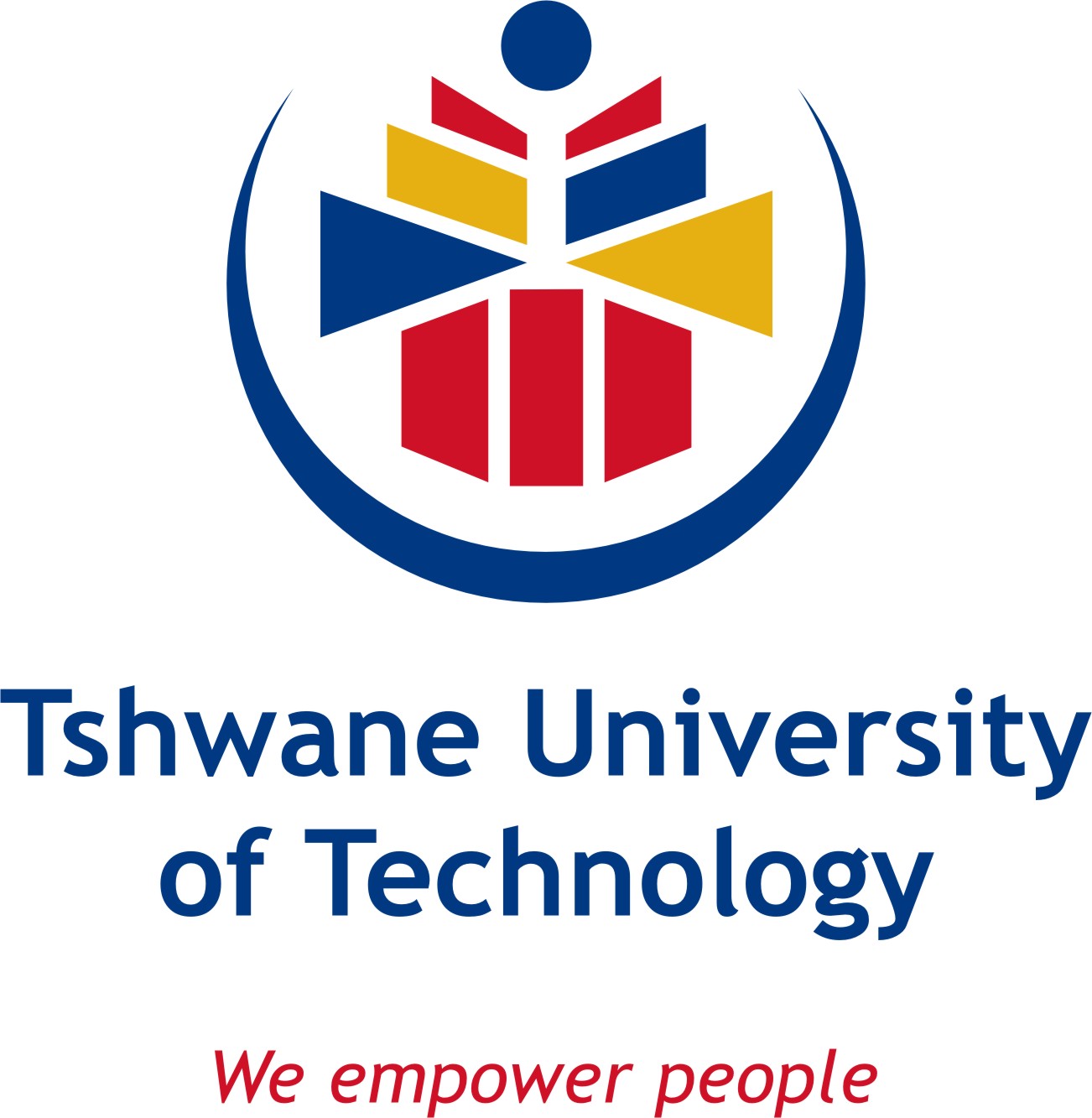


#### ETHICAL APPOROVAL

Faculty Committee for Research Ethics [FCRE-SCI]

*The TUT Senate Committee for Research Ethics is a registered Institutional Review Board (IRB 00005968) with the US Office for Human Research Protections (IORG# 0004997) (Expires 19 Jan 2014). Also, it has Federal Wide Assurance for the Protection of Human Subjects for International Institutions (FWA 00011501) (Expires 31 Jan 2014). In South Africa it is registered with the National Health Research Ethics Council (REC-160509-21).The FCRE-SCI is a subcommittee of the TUT Senate Committee for Research Ethics*

28 July 2016

Ref #: FCRE 2016/05/001 (3) (SCI)

Name: Moshoeshoe, RJ Student #: 207145343

Ms RJ Moshoeshoe

Department of Pharmaceutical Sciences

Dear Moshoeshoe,

Title: Assessing the legislative and policy framework for combating counterfeit medicines in South Africa

Ms RJ Moshoeshoe

Programme: M Tech: Pharmaceutical Sciences Supervisor: Prof D Katerere

Co-Supervisors: Dr GM Enslin

The amended M Tech proposal and all attachments has been reviewed by the Faculty of Science Committee for Research Ethics.

The Committee were satisfied that all their concerns were addressed in the amended proposal and attachments. It was mentioned that this was an excellent study.

The project is approved. The study may not commence on any of the sites before the parties involved give permission to access the site.

Annual review:

1. The formal ethics approval of all research projects need to be renewed on an annual basis.
2. The current ethics approval expiry date for this project is **31 December 2018**.
3. No research may continue after the ethics approval expiry date indicated on the formal Research Ethics Committee approval letter.
4. The Research Ethics Progress Report (electronic copy available at the following website:<http://www.tut.ac.za/Other/rninew/ResearchEthicsCommittees/Pages/default.aspx)> constitutes an application for such ethics approval renewal and must be submitted to the FCRE by **31, October 2018.**


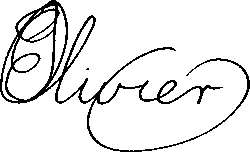
Kind regards

Chairperson: Prof EI Olivier

Faculty Committee for Research Ethics

Ref: FCRE 2016/05/001(3) (SCI)
