# Supplementary material for: An exploratory assessment of the legislative framework for combating counterfeit medicines in South Africa
Source: J Pharm Policy Pract. 2022 Jan 5;15:3. doi: 10.1186/s40545-021-00387-8 (PMC8730303; doi:10.1186/s40545-021-00387-8)
Supplement: Supplementary file 5 — Additional file 5. (addendum E): data collection tool for document analysis. [file 40545_2021_387_MOESM5_ESM.docx]

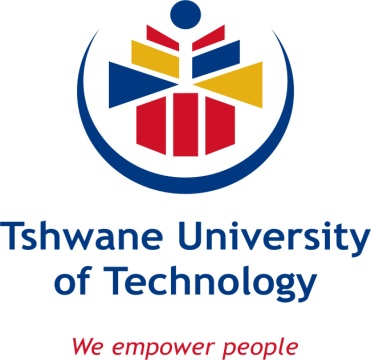


DEPARTMENT OF PHARMACEUTICAL SCIENCES

### FACULTY OF SCIENCE

**ASSESSING THE LEGISLATIVE AND POLICY FRAMEWORK FOR COMBATING COUNTERMEDICINES IN SOUTH AFRICA**

**DOCUMENT ANALYSIS DATA COLLECTION TOOLS**

# **SFs incident report analysis**

| Type of pharmaceutical product or case report | Type of offence | Manufacturer  Affected | Period of seizure | Seizure Units/net worth | Data Source |
| --- | --- | --- | --- | --- | --- |
|  |  |  |  |  |  |
|  |  |  |  |  |  |

#

# **Annual report analysis sheet**

| Information | 2011/12 | 2012/13 | 2013/14 | 2014/15 | 2015/16 | Comment |
| --- | --- | --- | --- | --- | --- | --- |
| Number of seizures |  |  |  |  |  |  |
| Reporting SFs |  |  |  |  |  |  |
| Training Specific to SFs |  |  |  |  |  |  |
| Law Enforcement Activities combating SFs |  |  |  |  |  |  |
| Awareness Campaigns on SFs |  |  |  |  |  |  |
