# Supplementary material for: An exploratory assessment of the legislative framework for combating counterfeit medicines in South Africa
Source: J Pharm Policy Pract. 2022 Jan 5;15:3. doi: 10.1186/s40545-021-00387-8 (PMC8730303; doi:10.1186/s40545-021-00387-8)
Supplement: Supplementary file 6 — Additional file 6. (addendum F): list of websites and databases. [file 40545_2021_387_MOESM6_ESM.docx]

| Agencies | Databases/type of document |
| --- | --- |
| Department of trade and industry | Annual reports, press releases and joint inspection reports  Website: [www.thedti.gov.za](http://www.thedti.gov.za) |
| Department of Health  South African Health Regulatory Products Authority | Annual reports, counterfeit medicine presentation slide.  Website:www.health.gov.za  Website: [www.sahpra.gov.za](http://www.sahpra.gov.za) |
| South African Revenue Service | Annual reports, press releases  Website:www.sars.gov.za |
| South African Police Service  South African Police Investigative Directorate | Annual reports  Website: [www.saps.gov.za](http://www.saps.gov.za)  Annual reports  Website: [www.gov.za](file:///C:\Users\rjmol\Desktop\SUBMISSION%20ARTICLE%202021\www.gov.za) |
| World Health Organisation | Website: [www.who.int](file:///C:\Users\rjmol\Desktop\SUBMISSION%20ARTICLE%202021\www.who.int) |
| World Trade Organisation | Website: [www.wto.org](file:///C:\Users\rjmol\Desktop\SUBMISSION%20ARTICLE%202021\www.wto.org) |
| United Nations Office of Drugs and Crime | Website: [www.unodc.org](http://www.unodc.org) |
| Pharmaceutical Security Institute | Website: [www.psi-inc.org](file:///C:\Users\rjmol\Desktop\SUBMISSION%20ARTICLE%202021\www.psi-inc.org) |
| National Prosecuting Authority | Website: [www.npa.gov.za](http://www.npa.gov.za) |
| INTERPOL | Website: [www.interpol.int](http://www.interpol.int) |
| Institute of Research Against Counterfeit Medicines | Website: [www.iracm.com](http://www.iracm.com) |
| Council of Europe | Website: [www.coe.int](file:///C:\Users\rjmol\Desktop\SUBMISSION%20ARTICLE%202021\www.coe.int) |
| Centre for Safe Internet Pharmacies | Website:www.safemedsonline.org |
