# Supplementary material for: An exploratory assessment of the legislative framework for combating counterfeit medicines in South Africa
Source: J Pharm Policy Pract. 2022 Jan 5;15:3. doi: 10.1186/s40545-021-00387-8 (PMC8730303; doi:10.1186/s40545-021-00387-8)
Supplement: Supplementary file 7 — Additional file 7. (addendum G): SA legislative framework. [file 40545_2021_387_MOESM7_ESM.docx]

| **Department** | **Legislation** | **Implications** |
| --- | --- | --- |
| Trade and Industry | Counterfeit Goods Act 37 of 1997 | The protection of trademark and copyright infringement |
| Health | Medicines and related Substances Act 101 of 1965 | Offences relating to sale of medicines (section 14), false labels & advertisements (Section 18), out of specifications, controlled substances and licenses (section 22C), sale and purchase by wholesalers (section 22H)  Penalties: - liable to a fine or imprisonment not exceeding 10 years |
| South African Revenue Services | Customs and Excise Act 91 of 1964  Section 113A, Section 5, Section 4(1) | -Customs duties & taxes  -Export and Import permits  -Search, seize and detain |
| South African Police Service | Criminal procedures act 51 of 1997 | Law enforcement, policing against criminal activities as well as counterfeited products |
| Department of Justice | Prevention of organised crime Act of 1998  Section 30, section 8(1) | Penalties: -  -Asset forfeiture  -Proceeds of crime: ≥R100 million or imprisonment not exceeding 30 years  Criminal gang activities: Imprisonment: Minimum: ≥3 years. Maximum: ≥ 8 years |
