# Supplementary material for: An exploratory assessment of the legislative framework for combating counterfeit medicines in South Africa
Source: J Pharm Policy Pract. 2022 Jan 5;15:3. doi: 10.1186/s40545-021-00387-8 (PMC8730303; doi:10.1186/s40545-021-00387-8)
Supplement: Supplementary file 8 — Additional file 8. (addendum H): Criteria for trustworthiness in the research process. [file 40545_2021_387_MOESM8_ESM.docx]

| **Criteria for trustworthiness** | **Strategies to enhance trustworthiness** | **Strategies to enhance trustworthiness applied to this research** |
| --- | --- | --- |
| **Truth value determines whether the researcher established confidence in the truth of the research findings.** | Credibility measures whether the research provides authentic representations of the respondents or their perceptions | The researcher engaged with respondents for 45 minutes and probed with questions for more in-depth explanations during interviews and follow up email and telephonic conversations after interviews. |
| **Applicability is the degree to which a study can be generalized.** | Transferability suggests that the study is reproducible in a similar context and research setting. | Data collection was done to a point of saturation where no new information emerged. A clear description of research methodology was provided that can be repeated to conduct similar studies. |
| **Consistency refers to the research yielding the same results if another study is done** | Dependability refers to protocol that the researcher provides of the research methodology that was followed. | The research methodology was clearly described and followed consistently. The researcher made reference to a body of literature and identifiable sources listed in a bibliography. |
| **Neutrality refers to the researcher being objective.** | Confirmability means that the researcher reduced bias as far as possible maintained and objectivity during the research. | The researcher recorded an honest reflection of respondents’ views and the supervisor verified all transcriptions. |
| **Authenticity refers to the extent to which the researcher describes the feelings or views of the respondents.** | Fairness implies that the study findings show the authenticity of work done and submitted. | The researcher ensured that respondents were knowledgeable on the subject of the study to ensure fairness. Respondents were asked to verify the responses given during the interviews afterwards. |

**Principles of trustworthiness and Anonymity test**
